# Protocol for the value of urodynamics prior to stress incontinence surgery (VUSIS) study: a multicenter randomized controlled trial to assess the cost effectiveness of urodynamics in women with symptoms of stress urinary incontinence in whom surgical treatment is considered

**DOI:** 10.1186/1472-6874-9-22

**Published:** 2009-07-21

**Authors:** Sanne AL van Leijsen, Kirsten B Kluivers, Ben Willem J Mol, Suzan R Broekhuis, Fred L Milani, C Huub van der Vaart, Jan-Paul WR Roovers, Marlies Y Bongers, Jan den Boon, Wilbert A Spaans, Jan Willem de Leeuw, Viviane Dietz, Jan H Kleinjan, Hans AM Brölmann, Eveline J Roos, Judith Schaafstra, John PFA Heesakkers, Mark E Vierhout

**Affiliations:** 1 Department of Obstetrics and Gynaecology, Radboud University Nijmegen Medical Centre, Nijmegen, the Netherlands; 2 Department of Obstetrics and Gynaecology, Academic Medical Centre Amsterdam, the Netherlands; 3 Department of Obstetrics and Gynaecology, Reinier de Graaf Gasthuis Delft, the Netherlands; 4 Department of Obstetrics and Gynaecology, Academic Medical Centre Utrecht, the Netherlands; 5 Department of Obstetrics and Gynaecology, Maxima Medical Centre Veldhoven, the Netherlands; 6 Department of Obstetrics and Gynaecology, Isala Klinieken Zwolle, the Netherlands; 7 Department of Obstetrics and Gynaecology, Gelre Hospital, the Netherlands; 8 Department of Obstetrics and Gynaecology, Ikazia Hospital Rotterdam, the Netherlands; 9 Department of Obstetrics and Gynaecology, Catharina Hospital Eindhoven, the Netherlands; 10 Department of Urology, Sint Franciscus Hospital Roosendaal, the Netherlands; 11 Department of Obstetrics and Gynaecology, VU University Medical Center, the Netherlands; 12 Department of Obstetrics and Gynaecology, Sint Antonius Hospital Nieuwegein, the Netherlands; 13 Department of Urology, Canisius Wilhelmina Hospital Nijmegen, the Netherlands; 14 Department of Urology, Radboud University Nijmegen Medical Centre, Nijmegen, the Netherlands

## Abstract

**Background:**

Stress urinary incontinence (SUI) is a common problem. In the Netherlands, yearly 64.000 new patients, of whom 96% are women, consult their general practitioner because of urinary incontinence. Approximately 7500 urodynamic evaluations and approximately 5000 operations for SUI are performed every year. In all major national and international guidelines from both gynaecological and urological scientific societies, it is advised to perform urodynamics prior to invasive treatment for SUI, but neither its effectiveness nor its cost-effectiveness has been assessed in a randomized setting.

The Value of Urodynamics prior to Stress Incontinence Surgery (VUSIS) study evaluates the positive and negative effects with regard to outcome, as well as the costs of urodynamics, in women with symptoms of SUI in whom surgical treatment is considered.

**Methods/design:**

A multicentre diagnostic cohort study will be performed with an embedded randomized controlled trial among women presenting with symptoms of (predominant) SUI.

Urinary incontinence has to be demonstrated on clinical examination and/or voiding diary. Physiotherapy must have failed and surgical treatment needs to be under consideration.

Patients will be excluded in case of previous incontinence surgery, in case of pelvic organ prolapse more than 1 centimeter beyond the hymen and/or in case of residual bladder volume of more than 150 milliliter on ultrasound or catheterisation.

Patients with discordant findings between the diagnosis based on urodynamic investigation and the diagnosis based on their history, clinical examination and/or micturition diary will be randomized to operative therapy or individually tailored therapy based on all available information.

Patients will be followed for two years after treatment by their attending urologist or gynaecologist, in combination with the completion of questionnaires.

Six hundred female patients will be recruited for registration from approximately twenty-seven hospitals in the Netherlands. We aspect that one hundred and two women with discordant findings will be randomized.

The primary outcome of this study is clinical improvement of incontinence as measured with the validated Dutch version of the Urinary Distress Inventory (UDI). Secondary outcomes of this study include costs, cure of incontinence as measured by voiding diary parameters, complications related to the intervention, re-interventions, and generic quality of life changes.

**Trial registration:**

Clinical Trials NCT00814749.

## Background

Stress urinary incontinence (SUI) is a frequently occurring problem. The Health Council of the Netherlands estimated that yearly 64.000 new patients, of whom 96% are women, consult their general practitioner because of urinary incontinence [1].

In all major national and international guidelines of professional organizations and authorities, it is advised to perform urodynamics prior to invasive treatment for SUI, e.g. the guidelines of the Dutch Urological Association, the Dutch Society of Obstetrics and Gynaecology, International Continence Society and European Association of Urology[2,3].

Usual care in the Netherlands for urinary incontinence in the general practitioners setting is an investigation by history, clinical examination and voiding diary. When there is no clear indication for urge incontinence or neurological disease, the patient will be referred for physiotherapy. In case there is no improvement the patient will subsequently be referred to the gynaecologist or urologist [4]. Patient history and clinical examination are important aspects of the assessment of patients that suffer from stress urinary incontinence. Patient history is quantified by using parameters based on validated questionnaires and a voiding diary for 1 to 2 days. The most common surgical therapy for SUI is the midurethral sling procedure of which the tension free vaginal tape (TVT) was first introduced. These slings have an average success rate of 90% [5].

Urodynamics do not generate major morbidity but are generally considered as unpleasant by the patients, and inhere a risk of urinary tract infections as high as 20% [6]. Urodynamics try to enhance the understanding of lower urinary tract functioning and reveal the underlying pathology that cause patients complaints. It is an extension of patient history and physical examination in an unphysiological setting.

The assumption is that the urodynamic setting is capable of making a distinction between several pathophysiological mechanisms causing the same micturition symptoms. If this holds true, the outcome of the available treatment options derived from the urodynamic based diagnosis would be better than treatment based on diagnosis made without urodynamics. However, the urodynamic investigations that differentiate between several types of SUI and specify for the type of operation, lack validation and predictive value in individual cases [7].

Moreover, since the introduction of easy to administer midurethral polypropylene slings, a simplified reasoning has found ground that states that every type of SUI is treated in the same way and therefore no urodynamic investigation would be needed.

The value of urodynamics has never been the specific subject of a randomized controlled trial [8]. Several studies have evaluated the value of urodynamics indirectly.

A retrospective cohort study on TVT has concluded that urodynamics do not have a predictive value on outcome after midurethral sling surgery [9]. In a randomized controlled trial on Burch compared with pubovaginal sling procedure, the predictive value of urodynamics was evaluated. Findings on urodynamic investigation did not seem to predict stress continence outcome [10].

The subjective and objective outcome of surgical intervention with or without preoperative urodynamic investigation has not been compared, with the exception of one retrospective study on colposuspension and one cohort study on TVT [11,12]. In these two studies no differences were found between the groups with and without urodynamics.

The strong conclusion from the Cochrane Review on the value of urodynamics was that a randomized trial is needed [13].

Thus, the role of urodynamics in the objectivation of SUI in women is nowadays questionable and very much under debate. In 25–30% of the women the symptom of SUI is not demonstrable probably due to the artificial situation during the investigation [5].

If urodynamics are not needed to diagnose types of SUI, this could be to prevent complications of surgery such as an overactive bladder. However in almost half of the patients with overactive bladder symptoms, there are no abnormal detrusor contractions visible on urodynamics [14]. On the other hand detrusor contractions during urodynamics that are regarded as the proof for overactive bladder complaints, can be seen in 20% of women without symptoms of an overactive bladder [15]. It is therefore questionable, whether it can predict the therapeutic effect as well as the risk of complications, like de novo urgency or aggravation of urgency and urinary retention [7,8].

The introduction of minimally invasive techniques for SUI therapy, such as the midurethral tension free tape procedure, has led to an enormous increase in the number of operations in the Netherlands. In 1999, 1,600 operations were performed. This number has increased to over 4,000 in 2003 and is expected to be 5,000 by today [16].

### Economic relevance

We estimate that two thirds of all women with symptoms of SUI who had urodynamics will proceed to surgery and one third will be given another non-surgical intervention which is usually medication. From this calculation it appears that for this indication urodynamics are performed approximately 7500 times per year in the Netherlands.

At expected costs of about 300 euro per test, urodynamics stand for 2.25 million euro health insurance charges.

In case urodynamics would be omitted, costs of possible extra complications are foreseen. We estimate a maximum of 10% additional urgency complaints [17,18]. Antimuscarinic treatment is available but has a record of only short time usage in almost all patients, and thus the costs are limited [19]. The other possible complication is urinary retention or voiding dysfunction. This occurs in less than 5% of patients after colposuspension, but is less in tension free midurethral slings and is supposed not to change with or without urodynamics [20]. This therefore will not have major financial impact on the outcome. Possible further additional costs are costs related to reoperation (repositioning of the sling estimated at 0.5–1%), and outpatients' costs.

In conclusion, it is likely that urodynamics do not have a role in the quality of care for women with SUI and that urodynamic testing in women with SUI is not cost-effective.

For this study our hypothesis was that there is no difference between outcome of surgery and individually tailored therapy in women with a discrepancy between urodynamic findings and findings from other investigations, such as history and clinical examination. In case of confirmation of this hypothesis, urodynamic investigation in women with predominant SUI could be safely omitted.

## Methods/design

The primary aim of the VUSIS study is to evaluate whether urodynamic testing is effective in patients with symptoms of SUI in whom surgical treatment is considered

**Figure 1:**
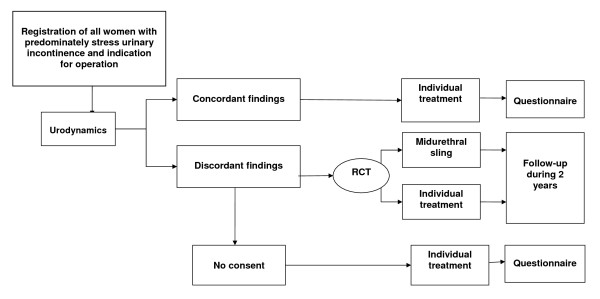
**Summary of trial design**.

The VUSIS is a multidisciplinary, multicentre diagnostic cohort study with a randomized controlled trial (RCT) embedded. All women with symptoms of predominantly SUI in whom surgical treatment is considered will undergo urodynamic investigation. Only women with a discordant finding compared to history and physical examination will be randomized. Patients with concordant urodynamics will be registered [see figure 1].

The study has been approved by the institutional review board of the Radboud University Nijmegen Medical Centre, in Nijmegen. Ethical approval for this study has been obtained on 02-10-2008, number 2006/197.

**Figure 2:**
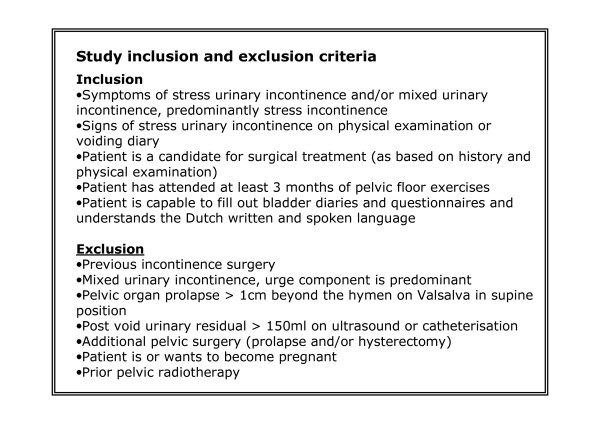
**Study inclusion and exclusion criteria**.

Eligible patients will be identified by gynaecologists and urologists from participating hospitals in the Netherlands [see Additional file 1]. All women presenting with SUI where conservative therapy (i.e. physiotherapy) has failed and surgical therapy is considered, will be asked for permission of registration in the study. SUI must have been demonstrated on physical examination and/or micturition diary. Patients will be excluded in case of previous incontinence surgery, in case of pelvic organ prolapse 1 centimeter beyond the level of the hymen and/or in case of residual bladder volume of more than 150 ml on ultrasound or catheterization [see figure 2].

In all patients the following items will be recorded at inclusion:

1. History and clinical examination

2. 48 h-Bladder (voiding and incontinence) diary

3. Validated Quality of Life questionnaires (Short Form 36, Euroqol 5D, Urinary Distress Inventory, Incontinence Impact Questionnair, Defecatory Distress Inventory)

4. Urinalysis for the detection of urinary tract infection

5. Residual urine measured by ultrasound

All women will undergo urodynamic investigation. Urodynamics will be performed according to International Continence Society standards and consists of free flow and measurement of residual, provocative filling cystometry with abdominal leak point pressure measurement, pressure flow study and a urethral pressure profilometry in rest and during stress[21]. The outcomes will be matched with urodynamic findings to assess the potentially useful parts of the urodynamic findings. Postoperative urodynamics is not part of the study.

**Figure 3:**
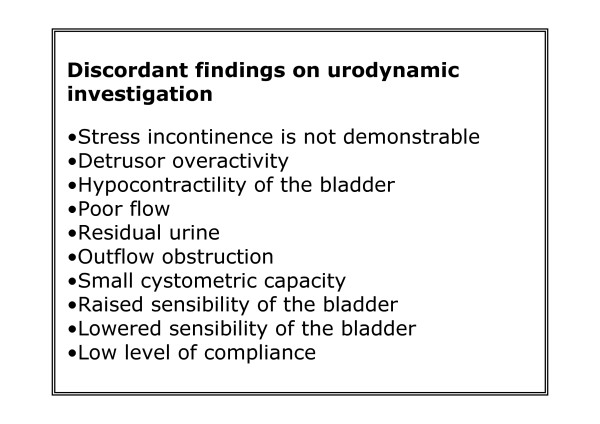
**Discordant findings on urodynamic investigation**.

When the result of the urodynamics does not confirm the history of SUI, or shows relative contra-indications for operation, the investigation is called discordant. Whether a test result is discordant is decided by the attending urologist or gynaecologist. Figure 3 demonstrates the various reasons for discordancy.

Women with discordant findings on urodynamics will be approached to participate in the randomized controlled part of the study.

After they have given informed consent, the patients with discordant test results at urodynamics are randomly assigned to the study or control group. In the study group, the decision for intervention will be based on history and clinical examination only, which will be surgical treatment; a midurethral sling. The choice for the kind of procedure is left to the discretion of the attending doctor. In the control group the decision for therapy will be based on history and clinical examination in combination with the result of the discordant findings on urodynamics. This includes medical treatment, prolonged physiotherapy, pessary treatment, but also surgical treatment can be one of the chosen therapies.

All women with SUI, who present at one of the participating hospitals, will be referred to a gynaecologist, urologist or a specifically appointed research nurse for counselling. Eligible women will receive an information sheet. Once women with discordant test results have given consent for the trial, they are randomized through a website, according to a computer-generated randomization sequence. Stratification will be applied for centre. Randomization will be 1:1 for operative treatment and individual treatment.

Patients in the RCT will be followed up from trial entry until the end of the study (anticipated average: two years). Follow-up is composed of the same items as recorded at inclusion.

After six weeks, six, twelve and twenty-four months patients will visit their attending gynaecologist or urologist. Physical examination will be performed and consists of detection of erosions, stress test for urinary leakage, and measurement of the residual volume. The patients will complete the questionnaires at all moments of follow-up.

After the intervention additional therapy is possible in both arms and will be recorded in the Case Record Form.

The cohort group will receive the same questionnaires once, at one year after the intervention.

### Patient outcome measures

The primary outcome of this study is clinical improvement of incontinence as measured with the validated Dutch version of the UDI.

Secondary outcomes of this study include costs, cure of incontinence as measured with voiding diaries, complications such as re-operation or overactive bladder symptoms, and quality of life.

The trial results will be incorporated in a diagnostic model to compare the current strategy (urodynamic evaluation in all patients) with the alternative strategy (immediate TVT surgery without urodynamic evaluation).

The study design will enable us to compare the costs and effects of the following strategies:

I. Immediate midurethral sling without preceding urodynamic evaluation.

II. Urodynamic evaluation in all patients and midurethral sling depending on urodynamic evaluation results.

III. Sequential or probabilistic combinations, based on the prior probability of the fact that the urodynamic evaluation will change management.

### Sample size considerations

The primary outcome of this study is the improvement of UDI at 12 months after baseline. The power calculation is performed using the non-inferiority assumption. The calculation is based on registration of 600 patients in the cohort study, of whom 200 will have discordant findings. When approximately 50% of the women give informed consent for randomization, 102 women with discordant findings will be randomized (51 to each group). As based on the non-inferiority assumption, the mean improvement in UDI in both groups is expected to be 35 with standard deviation of 10. A difference in mean improvement of 5 or less is considered as non-inferior (power 80% using one-sided testing at 0.05).

### Economic evaluation

For each patient, utilization of health care services will be recorded prospectively, using Case Record Forms, including urodynamic testing, surgery for SUI, re-operations, medical treatment for detrusor instability, care for urinary incontinence, and care for urinary retention. By multiplying these volumes of care with unit cost prices, direct medical costs incurred by SUI during the follow up period will be calculated for each patient. For unit cost prices, national guidelines will be used (CVZ, 2004). For costs of care for urinary incontinence and urinary retention, data from the literature will be used, converted to 2006 prices. We incorporated the health related quality of life questionnaire euroqol 5D in our study to be able to calculate QALYs (quality-adjusted life-years), which is a measure of health outcome. A QALY is the change in quality of life induced by the treatment multiplied by the duration of the treatment effect and it provides the number of QALYs gained. QALYs can then be related to medical costs to arrive at a final common denominator of cost/QALY. This parameter can be used to compare the cost-effectiveness of the treatment.

### Statistical analysis

Multivariate analysis of covariance with group, centre and the baseline covariate as independent variables will be used to estimate differences in improvement of the UDI after 12 months between the groups with 95% confidence intervals. As the UDI data are likely to be skewed, data will be log transformed prior to analysis. Other variables (i.e. IIQ) will be analyzed similarly.

### Time plan for the VUSIS study

Patient recruitment began in January 2009 and is planned to continue until January 2010. The follow-up has a duration of 24 months, so will continue until January 2012. The study is conducted in cooperation with several centers ensembled in the urogynaecology consortium. Most of the clinics have disposition over a research nurse, who attributes in administration and completion of the case record forms. All data are collected web based.

### Knowledge transfer

The outcome of the study will be important for the debate on the value of urodynamics. In the current international operative practice for SUI there is a huge increase in the number of operations. Hence the value of time consuming and costly urodynamic investigations should be very clear. The study is planned to be a starting point for a doctor's thesis. The results of our study will be submitted to different national and international scientific societies such as the International Continence Society and the International Urogynecologic Association, and is planned to be published in international scientific journals.

## Competing interests

The authors declare that they have no competing interests.

## Authors' contributions

KK and MV were responsible for the identification of the research question, and contributed to drafting of the study protocol. JH and BW have contributed to the development of the protocol and study design. All authors discussed the study design and commented on the protocol. SL was responsible for the drafting of this paper. All authors provided comments on the drafts and have read and approved the final version.

## Pre-publication history

The pre-publication history for this paper can be accessed here:



## Supplementary Material

Additional file 1 **Participating hospitals in the Netherlands**. This list gives an overview of the participating hospitals in the VUSIS study, all hospitals are located in the Netherlands. Click here for file

## References

[B1] Health council of the Netherlands Urinary Incontinence. The Hague: Health council of the Netherlands 2001; publication no 2001/12.

[B2] Groenendijk AG, Vervest HAM, Vaart CH van der, van Geelen JM (2004). Richtlijn Urine-incontinentie (Guideline Urinary Incontinence). Ned Vereniging voor Obstetrie en Gynaecologie.

[B3] Venema PL, van Geelen JM, Kil PJM (2003). Richtlijn Stressincontinentie bij de vrouw (Guideline Stress Incontinence in women). Nederlandse Vereniging voor Urologie.

[B4] Dutch College of General Practice (2009). Incontinence for urine. Utrecht.

[B5] Cardozo L, Khoury S, Wein A, Abrams P, (Eds) (2005). Incontinence, Plymouth UK.

[B6] Okorocha I, Cumming G, Gould I (2002). Female urodynamics and lower urinary tract infection. BJU Int.

[B7] Chapple CR, Wein AJ, Artibani W, Brubaker L, Haab F, Heesakkers JP (2005). A critical review of diagnostic criteria for evaluating patients with symptomatic stress urinary incontinence. BJU Int.

[B8] Heesakkers JP, Vriesema JL (2005). The role of urodynamics in the treatment of lower urinary tract symptoms in women. Curr Opin Urol.

[B9] Houwert RM, Venema PL, Aquarius AE, Bruinse HW, Kil PJ, Vervest HA (2009). Predictive value of urodynamics on outcome after midurethral sling surgery for female stress urinary incontinence. Am J Obstet Gynecol.

[B10] Nager CW, FitzGerald M, Kraus SR, Chai TC, Zyczynski H, Sirls L (2008). Urodynamic measures do not predict stress continence outcomes after surgery for stress urinary incontinence in selected women. J Urol.

[B11] Thompson PK, Duff DS, Thayer PS (2000). Stress incontinence in women under 50: does urodynamics improve surgical outcome?. Int Urogynecol J Pelvic Floor Dysfunct.

[B12] Schraffordt Koops SE, Bisseling TM, Heintz AP, van Brummen HJ, Vervest HA (2004). Are urodynamics a good tool for the prediction of succes of the TVT?. Abstract ICS/IUGA, Paris.

[B13] Glazener CM, Lapitan MC (2002). Urodynamic investigations for management of urinary incontinence in adults. Cochrane Database Syst Rev.

[B14] Digesu GA, Khullar V, Cardozo L, Salvatore S (2003). Overactive bladder symptoms: do we need urodynamics?. Neurourol Urodyn.

[B15] van Waalwijk van Doorn ES, Remmers A, Janknegt RA (1992). Conventional and extramural ambulatory urodynamic testing of the lower urinary tract in female volunteers. J Urol.

[B16] Vierhout ME (2005). [Increase in number of operations for stress urinary incontinence]. Ned Tijdschr Geneeskd.

[B17] Schraffordt Koops SE, Bisseling TM, Heintz AP, Vervest HA (2005). Prospective analysis of complications of tension-free vaginal tape from The Netherlands Tension-free Vaginal Tape study. Am J Obstet Gynecol.

[B18] Schraffordt Koops SE, Bisseling TM, Heintz AP, van Brummen HJ, Vervest HA (2005). Changes in irritative bladder symptoms after TVT. A prospective multicentre 3 year follow-up study with the aim of the urogenital distress inventory (UID-6) and incontinence impact questionnaire (IIQ-7). ICS abstracts Montreal.

[B19] Salvatore S, Khullar V, Cardozo L, Milani R, Athanasiou S, Kelleher C (2005). Long-term prospective randomized study comparing two different regimens of oxybutynin as a treatment for detrusor overactivity. Eur J Obstet Gynecol Reprod Biol.

[B20] Ward KL, Hilton P (2004). A prospective multicenter randomized trial of tension-free vaginal tape and colposuspension for primary urodynamic stress incontinence: two-year follow-up. Am J Obstet Gynecol.

[B21] Schafer W, Abrams P, Liao L, Mattiasson A, Pesce F, Spangberg A (2002). Good urodynamic practices: uroflowmetry, filling cystometry, and pressure-flow studies. Neurourol Urodyn.

