# Supplementary material for: Protocol for the value of urodynamics prior to stress incontinence surgery (VUSIS) study: a multicenter randomized controlled trial to assess the cost effectiveness of urodynamics in women with symptoms of stress urinary incontinence in whom surgical treatment is considered
Source: BMC Womens Health. 2009 Jul 21;9:22. doi: 10.1186/1472-6874-9-22 (PMC2722584; doi:10.1186/1472-6874-9-22)
Supplement: Additional file 1 — Participating hospitals in the Netherlands. This list gives an overview of the participating hospitals in the VUSIS study, all hospitals are located in the Netherlands. [file 1472-6874-9-22-S1.pdf]

Addendum 1. Participating hospitals in the Netherlands

1. Academic Medical Centre, Amsterdam
2. Alant Vrouw, Bilthoven
3. Canisius Wilhelmina Hospital, Nijmegen
4. Catharina Hospital, Eindhoven
5. Gelre Hospital, Apeldoorn
6. Hospital Zevenaar, Zevenaar
7. Ikazia Hospital, Rotterdam
8. Isala Klinieken, Zwolle
9. Laurentius Hospital, Roermond
10. Lievensberg Hospital, Bergen op Zoom
11. Martini Hospital, Groningen
12. Maxima Medical Centre, Veldhoven
13. Meander Medical Centre, Amersfoort
14. Medical Centre Haaglanden, the Hague
15. Medisch Spectrum Twente, Enschede
16. Onze Lieve Vrouwe Gasthuis, Amsterdam
17. Radboud University Nijmegen Medical Centre, Nijmegen
18. Reinier de Graaf Gasthuis, Delft
19. Rijnstate Hospital, Arnhem
20. Slingeland Hospital, Doetinchem
21. Sint Antonius Hospital, Nieuwegein
22. Sint Elisabeth Hospital, Tilburg
23. Sint Franciscus Hospital, Roosendaal
24. University Medical Centre, Groningen
25. University Medical Centre, Maastricht
26. VieCuri, Venlo
27. Zaans Medical Centre, Zaandam
